# *Fam151b*, the mouse homologue of C.elegans menorin gene, is essential for retinal function

**DOI:** 10.1038/s41598-019-57398-4

**Published:** 2020-01-16

**Authors:** Amy S. Findlay, Lisa McKie, Margaret Keighren, Sharon Clementson-Mobbs, Luis Sanchez-Pulido, Sara Wells, Sally H. Cross, Ian J. Jackson

**Affiliations:** 1 MRC Human Genetics Unit, Institute for Genetics and Molecular Medicine, University of Edinburgh, Edinburgh, UK; 2 Mary Lyon Centre, MRC Harwell, Oxford, UK; 3 Roslin Institute, University of Edinburgh, Edinburgh, UK

**Keywords:** Genetics, Eye diseases

## Abstract

*Fam151b* is a mammalian homologue of the *C. elegans menorin* gene, which is involved in neuronal branching. The International Mouse Phenotyping Consortium (IMPC) aims to knock out every gene in the mouse and comprehensively phenotype the mutant animals. This project identified *Fam151b* homozygous knock-out mice as having retinal degeneration. We show they have no photoreceptor function from eye opening, as demonstrated by a lack of electroretinograph (ERG) response. Histological analysis shows that during development of the eye the correct number of cells are produced and that the layers of the retina differentiate normally. However, after eye opening at P14, *Fam151b* mutant eyes exhibit signs of retinal stress and rapidly lose photoreceptor cells. We have mutated the second mammalian *menorin* homologue, *Fam151a*, and homozygous mutant mice have no discernible phenotype. Sequence analysis indicates that the FAM151 proteins are members of the PLC-like phosphodiesterase superfamily. However, the substrates and function of the proteins remains unknown.

## Introduction

The retina is the light sensitive tissue located in the posterior eye. Photoreceptor cells within the retina are responsible for converting light signals into electrical signals, and loss of these cells is a leading cause of blindness in humans. In the course of diseases such as retinitis pigmentosa (RP), rod and cone photoreceptor cells are slowly lost, beginning at the peripheral retina and moving centrally^1^. Multiple mutant genes in humans can cause RP, and many of these have counterpart mouse models^2^. In this paper we describe a novel mouse model of retinal degeneration which has early and rapid loss of photoreceptor cells due to loss of function of a largely uncharacterised gene. The International Mouse Phenotyping Consortium (IMPC) is a worldwide collaborative effort to generate lines of mice, each with a loss of function mutation in a single gene, and to comprehensively phenotype the lines^3,4^. The ultimate aim is to catalogue the function of every gene in the mouse genome in order to contribute to the understanding of fundamental mammalian biology and to generate models for human disease. Many institutions participate and follow an agreed pipeline for the phenotypic analysis of the mice, known as IMPReSS (International Mouse Phenotyping Resource of Standardised Screen). The analyses include growth, morphological, behavioural, metabolic and sensory phenotypes over a 15 week period, and followed post-mortem by examination of haematology, immunology, clinical chemistry and gross pathology. At 15 weeks of age mutant mice undergo slit lamp examination and ophthalmoscopy to investigate the gross morphology of the eye.

As part of this study a novel gene, *Fam151b*, was mutated and the mice analysed through the IMPC pipeline. *Fam151b* knockout mice were found to have abnormal retina morphology and no other observed phenotype. Here we follow up these findings with a detailed histological and functional analysis of this disease model. In addition we have mutated the paralogous gene, *Fam151a*. No retinal phenotype was observed in *Fam151a* mutant mice, nor was the *Fam151b* phenotype enhanced in double mutant animals.

*Fam151a* and *b* are mammalian homologues of the *C.elegans* gene *menorin* (*mnr-1*)^5^. This gene was identified as affected in mutant worms with disrupted dendritic branching in somatosensory neurons; the 90° menorah-like branch pattern observed in control worms was lost in mutants. Mutations in other genes that result in a similar phenotypes, and subsequent biochemistry, identified SAX7 (the *C. elegans* homologue of L1CAM) and the leucine rich repeat transmembrane receptor, DMA*-1*, as forming a genetic and molecular complex with MNR-1 which is necessary for dendritic branching.

## Results

### Menorin, FAM151A and B are members of the GDPD/PLCD superfamily

**Figure 1:**
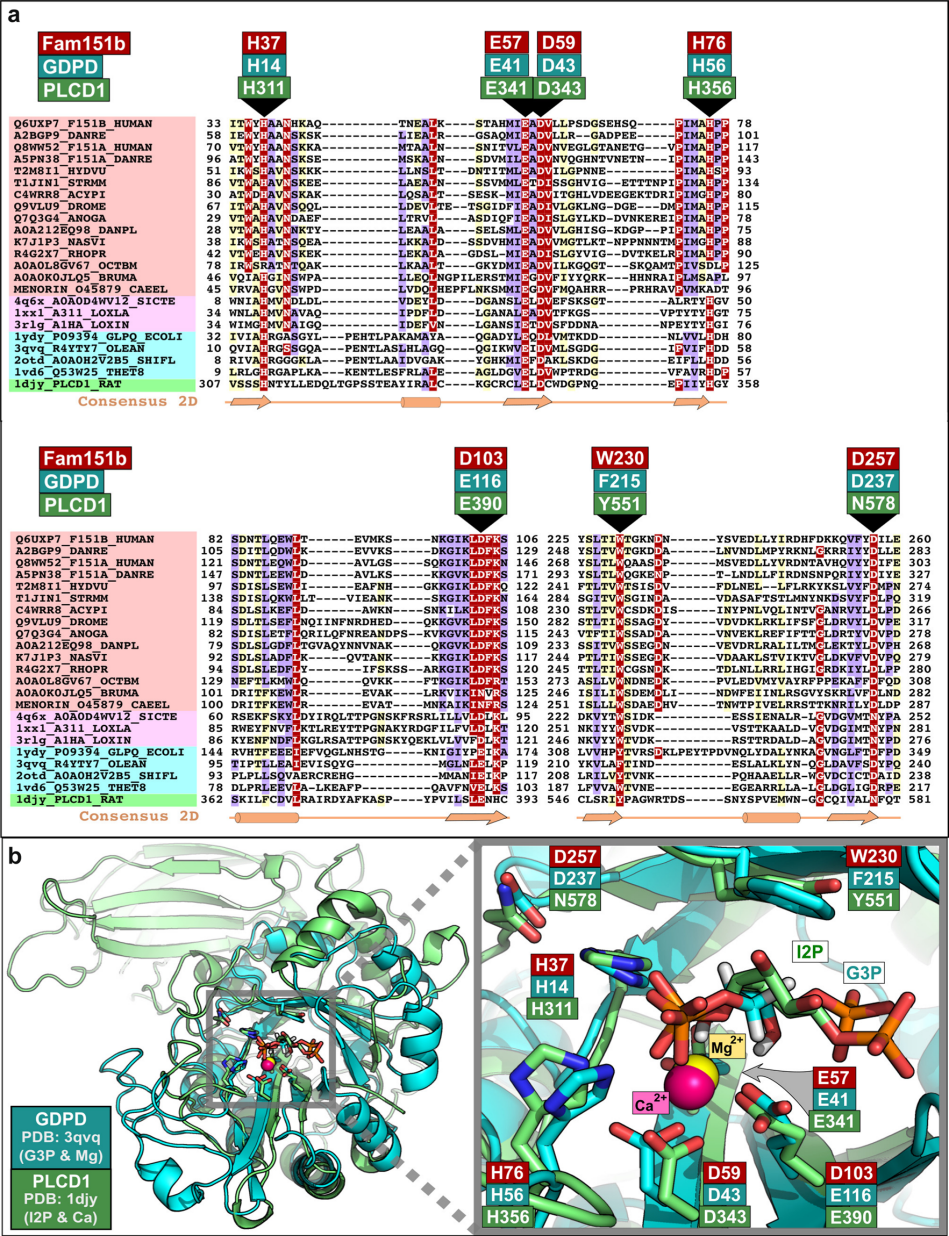
(**a**) Multiple sequence alignment (MSA) of representative members of FAM151 family and PLC-like phosphodiesterases. This MSA was generated with the program T-Coffee^28^ using default parameters and slightly refined manually. The final superfamily alignment was generated using a combination of profile-to-profile comparisons^26^ and sequence alignments derived from structural superimpositions using DALI^29^, for those families whose tertiary structure is known, such us: Phospholipase D toxins (PDB-IDs: 4Q6X, 1XX1, and 3RLG), GDPD enzymes (PDB-IDs: 1YDY, 3QVQ, 2OTD, and 1VD6) and PLC (Phosphoinositide phospholipase C) enzyme (PDB-ID:1DJY). Families are indicated by coloured background to the left of the alignment: FAM151, Phospholipase D toxins, GDPD and PLC enzymes are indicated in red, pink, blue and green, respectively. The limits of the protein sequence conserved regions included in the alignment are indicated by flanking residue positions. Secondary structure predictions^30^ were performed for the FAM151 family and these are consistent with X-ray determined secondary structures of the PLC-like phosphodiesterases. Alpha-helices and beta-strands are indicated by cylinders and arrows, respectively. The alignment was presented with the program Belvu using a colouring scheme indicating the average BLOSUM62 scores (which are correlated with amino acid conservation) of each alignment column: red (>3), violet (between 3 and 1.5) and light yellow (between 1.5 and 0.5)^31^. Sequences are named according to their UniProt identification. (**b**) Structural superimposition of two members of the PLC-like phosphodiesterases superfamily: GDPD and PLCD1. Active site residues are labelled and coloured according to their reference protein sequences: human FAM151B, GDPD (PDB-ID: 3QVQ) and PLCD1 (PDB-ID:1djy) in red, blue and green, respectively. Structures are presented using Pymol (http://www.pymol.org).

We analysed the amino acid sequence of FAM151B to identify similarities with proteins of known function. A search of the UniRef 50 database^6^ shows the FAM151 family to be widely distributed in animals, including *C.elegans*, as noted above, and *Drosophila*, and is annotated in Pfam as containing the domain of unknown function (DUF) 2181. We used the remote homology detection server HHPred^7^ using a profile from multiple FAM151 alignments to find more divergent homologues. HHPred searches with FAM151 family matched the Phospholipase D domain of a spider (*Sicarius terrosus*) toxin (PDB-ID: 4Q6X) with a highly significant E-value of 1.4 × 10^−5^ ^8^. Moreover, in support of this, the next most statistically significant matches were to additional members of the Phospholipase D and glycerophosphodiester phosphodiesterase (GDPD) families (Fig. 1). These proteins are all members of the PLC-like phosphodiesterase superfamily of enzymes. PLC-like phosphodiesterases contain a TIM barrel fold, first described in triosephosphate isomerase but found in many proteins, and contain an evolutionarily conserved active site (Fig. 1) crucially also conserved in menorin and FAM151A and B. These different enzymes hydrolyse phosphodiester bonds of a large number of different substrates, ranging from Glycosylphosphatidylinositol (GPI) protein anchors (in GDPD family) to sphingolipid and lysolipid (in Phospholipase D spider toxins^8–11^.

FAM151B has a single DUF2181 domain, as does menorin. In FAM151A the DUF domain is duplicated, but the critical active site residues in the C-terminal domain are missing suggesting that this second domain lacks enzymatic activity. Most vertebrates have orthologues of both FAM151A and B, but *Medaka* is one exception and appears to have only FAM151B.

The expression patterns of the two *Fam151* paralogues are quite different. *Fam151a* expression in the mouse is undetectable in most tissues, but is highly expressed in the intestine, kidney and spleen. In contrast, *Fam151b* is found expressed at low levels in most tissues including the retinal pigmented epithelium (RPE), retina, iris, ciliary body, lens and cornea. (BioGPS: www.biogps.org).

### *Fam151b* knockout mice exhibit early and rapid photoreceptor loss

**Figure 2:**
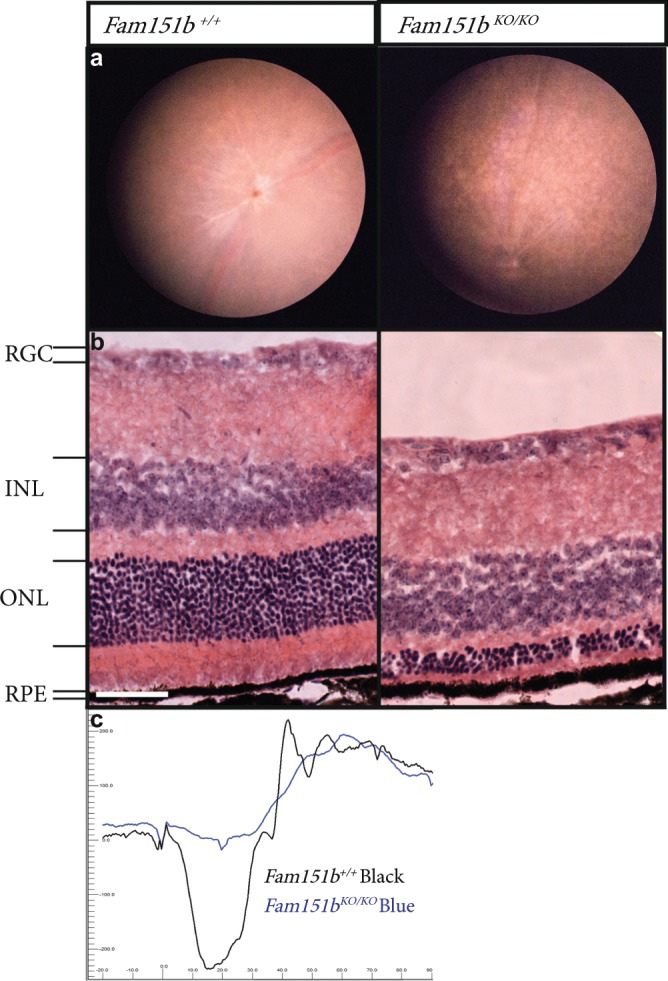
Adult *Fam151b*^*KO/KO*^ mice have extensive retinal degeneration. (**a**) Comparison of 11 week old *Fam151b*^*KO/KO*^ mice and their wild-type littermates fundal images shows an uneven appearance on the Fam151b^KO/KO^ mouse retina indicative of abnormal retinal morphology. Histological analysis (**b**) shows that this was due to a loss of the outer nuclei layer, in which the nuclei of photoreceptor cells are located. The outer segments are shortened. This loss of nuclei is directly related to a loss of photoreceptor cells. (**c**) Functional analysis of the retina through ERG traces shows a loss of scotopic a-wave, produced from photoreceptor cells response to a light stimulus, *Fam151b*^*KO/KO*^ (blue line) compared with wild type littermates (black line). RGC = Retinal Ganglion Cells, INL = Inner Nuclei Layer, ONL = Outer Nuclei Layer, RPE = Retinal Pigment Epithelium. Scale bar = 50 µm.

*Fam151b* mutant mice containing a LacZ reporter inserted into intron 2 and a deletion of exon 3 (*Fam151b*^*tm1b(EUCOMM)Hmgu*^ hereafter *Fam151b*^*KO*^) were derived as described in the Methods. The insertion includes a polyadenylation site, and the deletion results in any mRNA from which the insertion has been spliced out having a frameshift and termination of translation 15 codons further. Phenotyping homozygous mutant mice through the IMPReSS pipeline identified a degenerative retinal phenotype at 15 weeks, characterised by patchy pigmentation of the retina, compared to the evenly coloured pink retina of control mice. We examined homozygous mice earlier, at 11 weeks, and also observed a patchwork pattern from fundal imaging indicative of retinal degeneration, not present in wild type litter mates (Fig. 2a). Histological analysis of the eyes revealed a severe reduction in the length of the outer segments of the photoreceptors, and in the number of the nuclei of the photoreceptor cells, found in the outer nuclear layer, indicating a substantial loss of photoreceptors. (Fig. 2b). The other retinal layers appear to be unaffected. To test for retinal function we carried out electroretinogram (ERG) analysis on the mutant mice, which confirmed a loss of photoreceptor function, shown by a greatly reduced scotopic a-wave (Fig. 2c).

**Figure 3:**
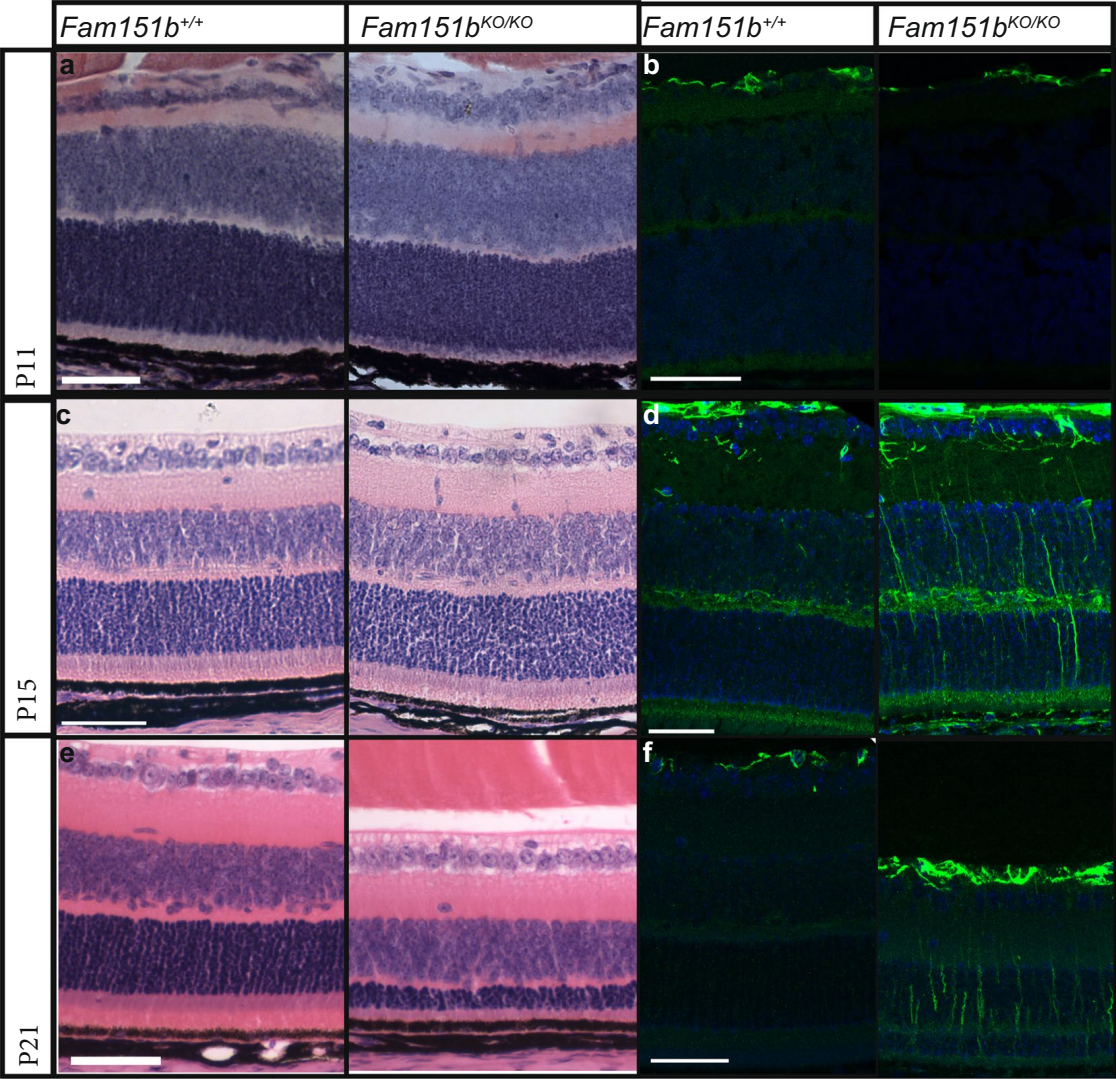
Histological analysis of disease progression in *Fam151b*^*KO/KO*^ mice. Haematoxylin and eosin staining of P11 (**a**), P15 (**c**) and P21 (**e**) *Fam151b*^*KO/KO*^ retinal sections compared with *Fam151b*^*+/+*^ littermates shows the loss of photoreceptor nuclei by P21. At P11 and P15 mutant mice sections show comparable numbers of nuclei to wild-type. All other retinal cell layers seem unaffected by the loss of *Fam151b* expression. GFAP staining in green shows that at P11 (**b**) Fam151b^KO/KO^ sections have similar staining in the RGC layer as wild-type littermates. However, by P15 (**d**) and continuing on to P21 (**f**) mutant sections exhibit a clear upregulation of GFAP indicative of retinal stress. Scale bar = 50 µm.

We analysed *Fam151b* mutant and control eyes at several ages to determine when the loss of photoreceptor cells occurred. Eyes were taken at postnatal day 11 (P11) and examined by histology (Fig. 3). Mutant mice at this age had comparable numbers of nuclei in the outer nuclei layer to wild type littermates, and the remainder of the retina also appears normal. The mutant eyes appear to develop normally prior to eye opening at P14. We stained the retinal sections to label glial fibrillary acidic protein (GFAP), a widely used marker of retinal stress^12^, and found no upregulation of this protein at P11.

We compared *Fam151b*^*KO/KO*^ and wild type littermates at P15. As shown in Fig. 3, mutant mice still have comparable numbers of photoreceptor nuclei to wild type littermates. However, at this stage, there is an upregulation of GFAP expression, indicative of retinal stress. By P21 *Fam151b*^*KO/KO*^ mice have a dramatically reduced outer nuclei layer (Fig. 3) indicating a loss of photoreceptor cells along with GFAP upregulation.

**Figure 4:**
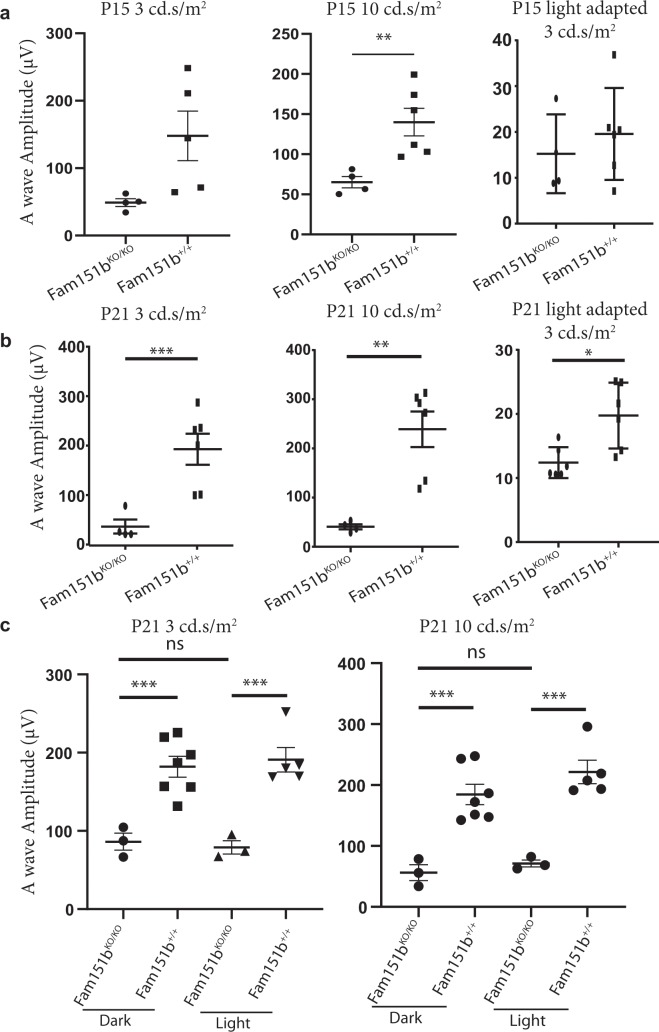
ERG a-wave amplitudes plotted for comparison. (**a**,**b**) Compares P15 (**a**) and P21 (**b**) mutant mice with wild-type littermates, using 3, 10 cd.s/m^2^ light stimuli on dark adapted mice followed by 3 cd.s/m^2^ light stimuli on light adapted mice (n = 4 (mutant), 6 (wild type)). There is a significant reduction in a-wave amplitude in the Fam1511bKO/KO mice compared to wild-type controls except at P15 for 3 cd.s/m^2^ light stimulus in both dark and light adapted mice. (**c**) Comparison of P21 mice kept in either darkened cage conditions or a normal 12 hour light cycle (n = 3 (mutant dark), 7 (wild type dark), 3 (mutant light), 5 (wild type light)). **p < 0.01, ***p < 0.001 by unpaired t-test with Welch’s correction.

We performed ERG on P15 mice to assess whether the photoreceptors present have any function prior to their degeneration. As shown in Fig. 4a, *Fam151b*^*KO/KO*^ mice exhibit a significantly reduced scotopic (dark adapted) a-wave when stimulated with a high intensity light source (10 cd.s/m^2^) although at standard light intensity, in both dark adapted and light adapted eyes, (3 cd.s/m^2^) whilst the a-wave amplitude appears reduced it does not reach significance at p = 0.0534 and 0.487931 for dark and light adapted eyes respectively. This suggests that some of the photoreceptors are able to respond to a light stimulus, however the majority cannot and thus produces a lower a-wave amplitude. This may mean that the photoreceptor cells develop and can function normally but degenerate very quickly after eye opening. By P21 there is little to no a-wave amplitude when stimulated with both 10 cd.s/m^2^ and 3 cd.s/m^2^ flashes (Fig. 4b). This is replicated in the light adapted response showing that cones are also being lost.

### The retinal pigment epithelium of *Fam151b*^*KO/KO*^ mice appears healthy

**Figure 5:**
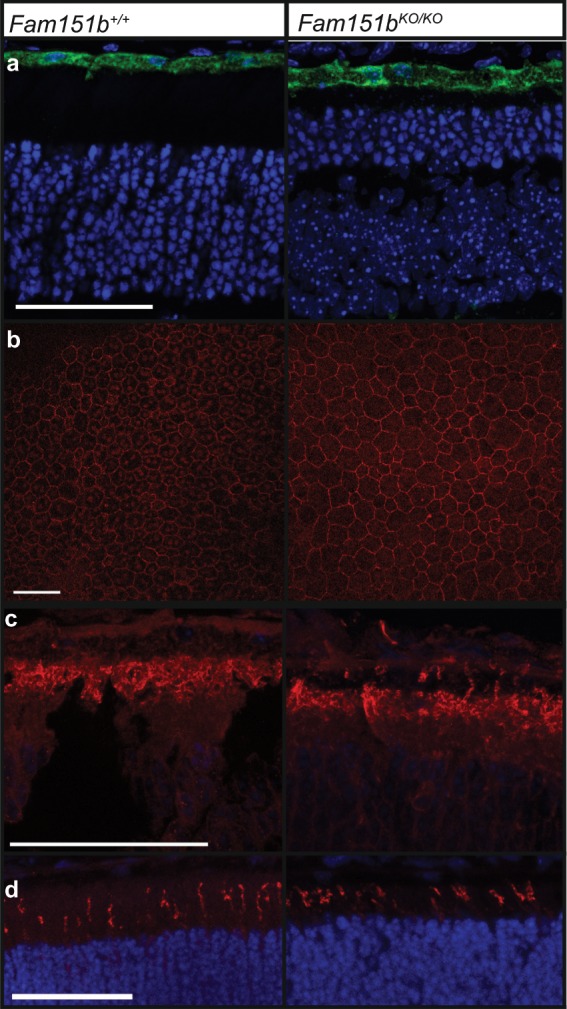
RPE health was assessed by examining typical RPE markers. (**a**) RPE65 showed correct localisation to the RPE in retinal sections of P15 mice and (**b**) ZO-1 localised to tight junctions when observed on flat mount RPE in both P30 *Fam151b*^*KO/KO*^ and wild type. The correct localisation of light sensitive opsins is essential for the proper function of photoreceptors, in P15 *Fam151b*^*KO/KO*^ mutant eyes both rhodopsin (**c**) and m-opsin (**d**) localise to the outer segments of photoreceptor cells as expected. Scale bar 50 µm.

The retinal pigment epithelium (RPE) is important for the survival of photoreceptor cells as it provides support and recycles components of photoreceptor specific pathways^13^. The early and rapid loss of photoreceptor cells seen in the *Fam151b*^*KO/KO*^ mice may be indicative of an RPE defect, so we examined two key RPE markers. Both RPE65 and ZO-1 are typical RPE and epithelial cell markers respectively and are both present and localised as expected. RPE65 is a membrane-associated RPE specific enzyme necessary for the generation of 11-cis-retinol in the retinoid cycle. Antibody staining on sections of 2 month old retinal tissue showed clear expression and localisation to the RPE layer in mutant mice (Fig. 5). ZO-1 localises to the tight junctions between cells. ZO-1 antibody staining on 2 month old flattened whole RPE, from which the rest of the retina had been carefully removed, enabled us to view the localization of ZO-1 at the tight junctions in both mutant and control RPE (Fig. 5).

P15 retinal sections stained for the light sensitive rod and cone opsins, rhodopsin and M/L-opsin respectively, showed that in mutant *Fam151b*^*KO/KO*^ opsins localised to the outer segments of photoreceptor cells (Fig. 5).

### Photoreceptor loss is not due to light toxicity

Mice open their eyes at about P14, just before we observed increased GFAP expression and reduced retinal function, raising the possibility that photoreceptor function may be lost due to light toxicity. To test this we removed pups, along with their mothers, from a 12 hour light/dark cycle and placed them in a constant dark environment for 1 week from P13, about one day prior to eye opening, until P20 and immediately thereafter analysed their ERG. We compared ERGs from *Fam151b*^*KO/KO*^ mice kept in a dark environment with wild-type littermates kept in the same environment and with *Fam151b*^*KO/KO*^ mice and wild-type littermates kept in a normal 12 hour light/dark cycle (Fig. 4c). The mutant mice maintained in the dark exhibited similar photoreceptor responses to both standard and high intensity flashes, compared with *Fam151b*^*KO/KO*^ kept in a normal light cycle. The ERG responses of wild-type mice kept in the dark indicated that the absence of light over this period of development did not affect their photoreceptor function.

### *Fam151b*^*KO/KO*^ mice do not show disrupted nerve patterning

**Figure 6:**
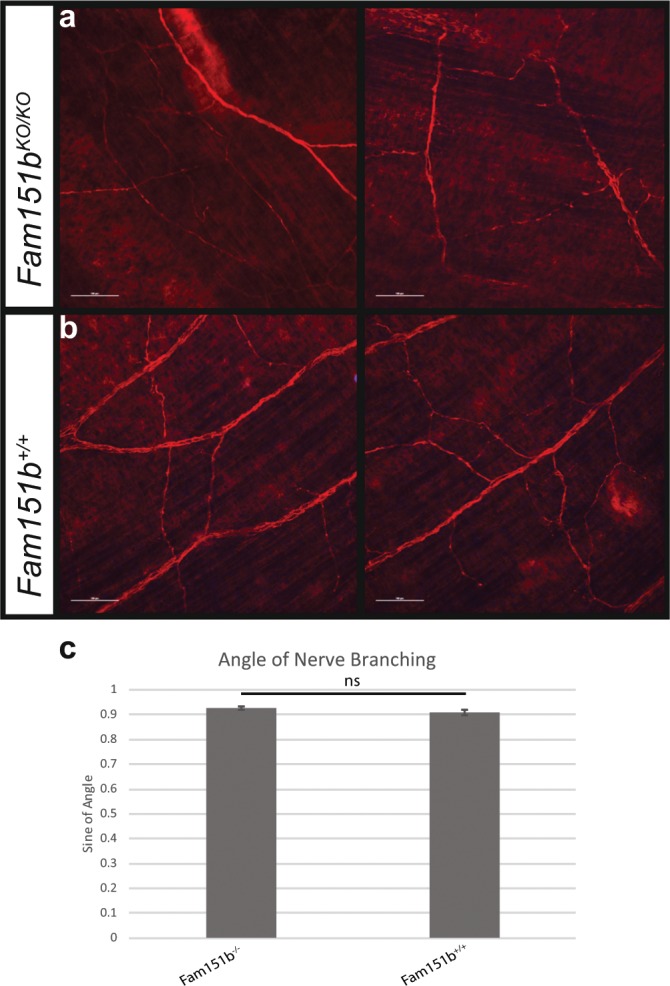
Nerve branching in the skin was normal in *Fam151b*^*KO/KO*^ mice. Neurofilament staining was done on flat mount skin from embryonic day 16.5 (E16.5) Fam151b^KO/KO^ mice (**a**) and wild type littermates (**b**) in order to analyse the branching patterns of the nerves. (**c**)The angle of branching nerves was measured and the sine of the angle was used for analysis. No significant difference was found between Fam151b^KO/KO^ mice and their wild-type counterparts (*p* = 0.1869, unpaired t-test, n = 6). Scale bar 100 µm.

Given the role of the homologue of *Fam151b*, *Menorin*, in *C. elegans* we looked at the patterning of nerves in embryonic skin by neurofilament staining. Developing nerves within the skin were visualised and their branch angles measured. No significant difference was found between *Fam151b*^*KO/KO*^ mice and their wild-type littermates. (Fig. 6).

### *Fam151a* mutant mice

**Figure 7:**
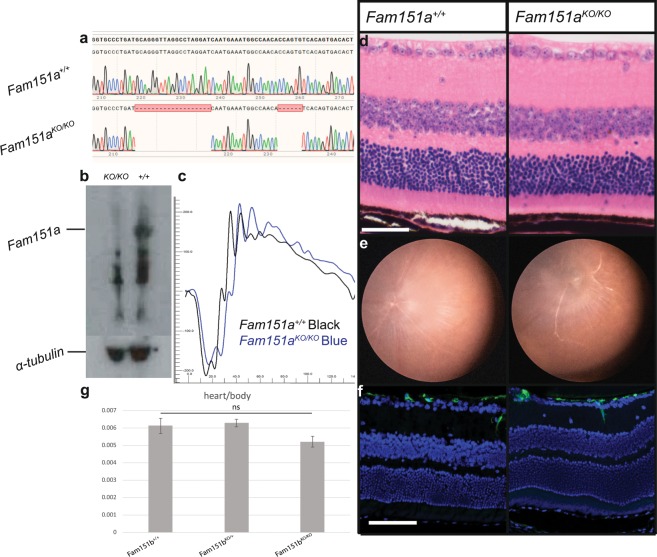
*Fam151a*^*KO/KO*^ mice were aged to one year to observe whether they exhibit late onset retinal degeneration not picked up on at the 15 week fundal examination by IMPC. (**a**) We used CRISPR Cas-9 to produce the *Fam151a*^*KO/KO*^ mice and here we show the two separate deletion caused in exon 1 of the gene (bottom line) compared to wild type sequence (top line). (**b**) Western blot showing loss of Fam151a protein in kidney samples of Fam151a^KO/KO^ mice compared to Fam151a^+/+^, band at 66 kDa highlighted. Alpha-tubulin used as a loading control. Functional analysis by ERG (**c**) shows comparable response to a light stimulus as that seen in wild-type littermates. (**d**) Histology and (**e**) fundal imaging both show normal retinal morphology, no decrease in photoreceptor layer thickness was seen and fundal images showed an even and healthy appearance. (**f**) GFAP staining showed no upregulation, indicative of no retinal stress. (**g**) Comparison of heart weight in relation to body weight between *Fam151b*^*KO/KO*^ mutant mice and wild-type littermates. No significant difference was found by t-test (*p* = 0.1275). Scale bar = 50 µm.

We asked if mutations in the paralogous gene, *Fam151a*, had any detectable phenotype and in particular if they resulted in a similar mutant retinal phenotype. Previously, mice homozygous for a targeted mutation in *Fam151a* had been bred and phenotyped as part of the IMPC effort and no eye defect was reported at 15 weeks of age. To confirm this, using CRISPR, we generated several mice carrying *Fam151a* mutations. We selected one, which had a complex double deletion of 19 and 6 bp, flanking a 16 bp section in exon 1, predicted to cause a premature stop codon in exon 2 (Fig. 7a) which showed a loss of Fam151a protein in a Western performed on kidney samples (Fig. 7b), and bred homozygous mutant mice which were aged alongside wild-type and heterozygous littermates.

At one year, ERG analysis of the mutant mice found no decrease in retinal response to a light stimulus; both a-waves and b-waves were similar to those observed in the wild-type litter mates (Fig. 7c). Gross retinal morphology also appeared normal on fundal imaging, and histological analysis showed no loss of photoreceptor cells, or increased presence of GFAP staining (Fig. 7d–f).

During the IMPC analysis of the *Fam151a* mutant mice 1 of 16 homozygous mutant mice was described as having abnormal heart morphology; an enlarged heart. We measured the heart weight relative to body weight of our *Fam151a* mutant mice and found no significant difference between *Fam151a*^*KO/KO*^ mutants and their wild-type and heterozygous litter mates (Fig. 7g).

### *Fam151a/Fam151b* double mutant mice do not exhibit a worsened phenotype

**Figure 8:**
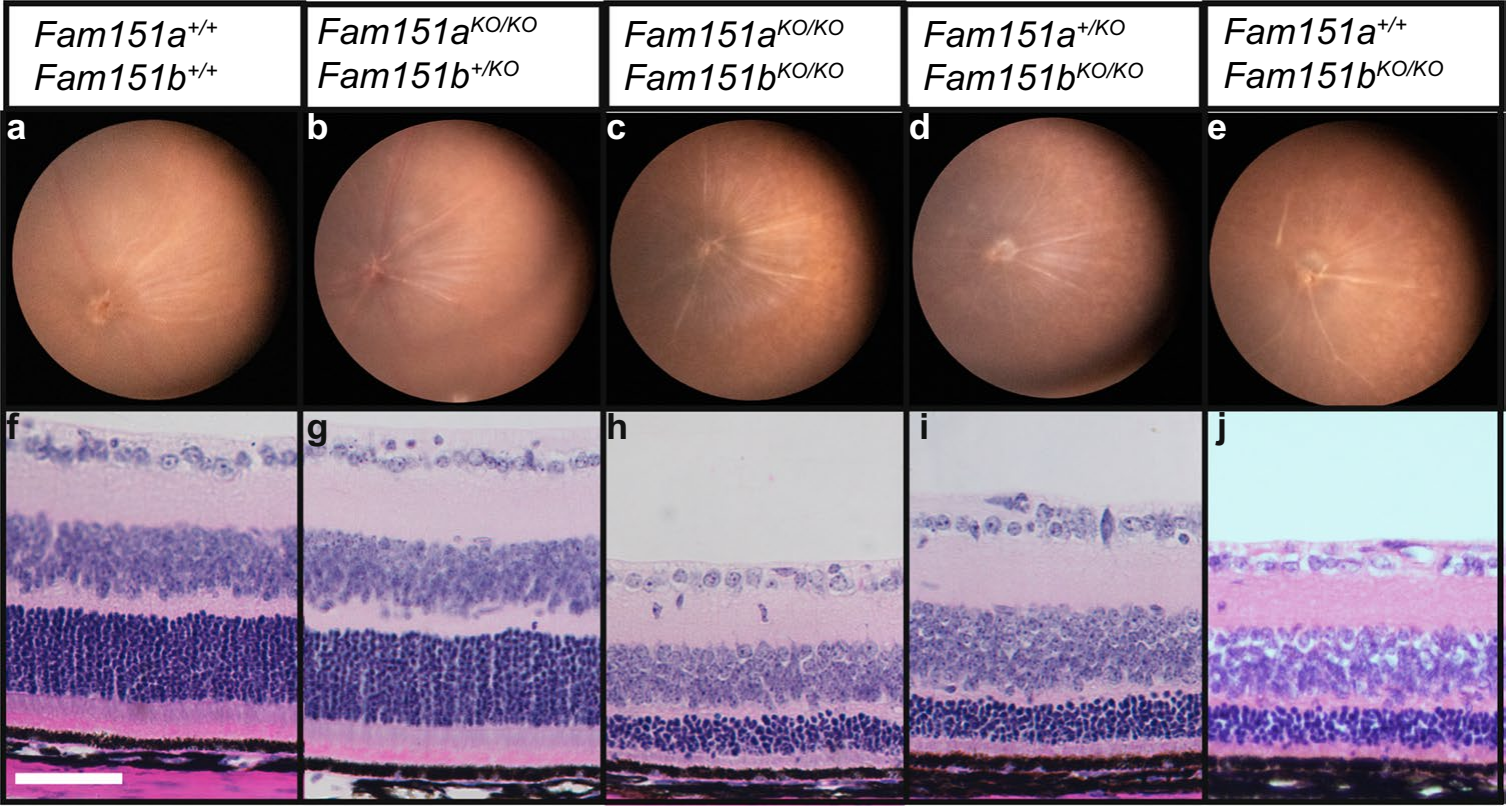
Loss-of-function of *Fam151a* does not exacerbate the *Fam151b*^*KO/KO*^ mutant eye phenotype. *Fam151a*^*KO/KO*^*Fam151b*^*KO/KO*^ double mutants were analysed alongside control littermates. Fundal images (**a**–**e**) and histology (**f**–**j**) were performed on P21 mice. The retinal morphology and loss of photoreceptor cell nuclei of *Fam151b*^*KO/KO*^ mice was unaffected by Fam151a status. Scale bar 50 µm.

We asked if the paralogous *Fam151* genes compensated for each other in viability or gross phenotype in the mutant lines. To address this *Fam151a* and *Fam151b* mutant strains were bred together to produce *Fam151*^*KO/+*^*/Fam151b*^*KO/+*^ double heterozygotes which were then used in matings and offspring were observed at Mendelian ratios indicating that the mice were viable. We also observe the expected number of each genotype at weaning (Chi-squared (8 degrees of freedom) p = 0.3712) (Table 1). We examined the gross retinal morphology of the retinas through fundal imaging at 3 weeks. Double homozygous mutants showed the same degree of retinal degeneration when compared to *Fam151a*^*+/+*^ *Fam151b*^*KO/KO*^ littermates, which we confirmed with histology, showing no increase in the loss of photoreceptor cells in the double homozygous mice (Fig. 8). Mice were also maintained until one year of age and no abnormal phenotype, other than retinal degeneration, was observed.

**Table 1:** Observed and expected offspring number from *Fam151a*^*KO/+*^*Fam151b*^*KO/+*^ double heterozygote matings.

Genotype	Observed	Expected
*Fam151a*^*+/+*^ *Fam151b*^*+/+*^	5	3.6
*Fam151a*^*+/+*^ *Fam151b*^*KO/+*^	10	7.25
*Fam151a*^*+/+*^ *Fam151b*^*KO/KO*^	5	3.6
*Fam151a*^*KO/+*^ *Fam151b*^*+/+*^	7	7.25
*Fam151a*^*KO/+*^ *Fam151b*^*KO/+*^	13	14.5
*Fam151a*^*KO/+*^ *Fam151b*^*KO/KO*^	8	7.25
*Fam151a*^*KO/KO*^ *Fam151b*^*+/+*^	2	3.6
*Fam151a*^*KO/KO*^ *Fam151b*^*KO/+*^	2	7.25
*Fam151a*^*KO/KO*^ *Fam151b*^*KO/KO*^	6	3.6

## Discussion

Loss of photoreceptors is a leading cause of blindness, understanding the genetic causes and pathology of the cell death is essential for developing therapeutics and possibly for developing ways of preventing the disease. Mouse models are an excellent resource in this area of research as they often recapitulate the human disease and therefore can be used to test the effectiveness of treatments and preventative strategies.

Here we show that the *Fam151b* gene is essential for photoreceptor survival in the mouse. Retinal function also appears to be compromised with a reduced scotopic ERG response on eye opening in mice deficient for *Fam151b*. With such an early phenotype a diminished production or localisation of light sensitive proteins might be expected. However, rhodopsin and M-opsin staining in photoreceptors is still present and localised to the outer segments. These proteins are still observed at later stages, at week 11 two layers of photoreceptors remain with correct localisation of opsins, indicating that after the initial surge of cell death the surviving cells retain some aspects of normal cellular architecture. To date no patients with mutations in either *FAM151* paralogues have been described. Clear loss of function mutation in *FAM151B* are on the whole rare, and show a pattern similar to other retinal disease-causing genes. Light is well known to cause or further exacerbate retinal degeneration^14–17^. Photoreceptors must constantly renew essential pathway components and remove toxic products. Because of this and due to the timing of the retinal degeneration, we investigated whether light caused photoreceptor loss in the *Fam151b*^*KO/KO*^ mice and found this not to be the case. Mice kept in a dark environment from before their eyes were open showed a similar rate of degeneration. It is assumed that prior to eye opening some light may leak through the eyelids, but as no GFAP staining was observed in P11 retinas this is unlikely to be the cause of degeneration.

The molecular function of menorin/FAM151A and B is unknown. The *C.elegans* protein, SAX-7, interacts with menorin to control the dendritic branching of sensory neurons^5^. Mutants of the mammalian homologue of *Sax-7*, *L1CAM*, have been described as having abnormal axon guidance and retinal ganglion cells, as well as other neuronal problems^18–21^. This phenotype has similarities to that seen in *C. elegans* mutants, and has implicated L1CAM in the precise guidance of axons. However this differs from the phenotype we have observed in *Fam151b*^*KO/KO*^ mice. Furthermore, the branch pattern of nerves located in the skin was analysed and no abnormality was observed in mutant mice (Fig. 6). In addition, loss of dendritic complexity in humans has been linked to behavioural defects, autism and schizophrenia^22,23^. Analysis of the *Fam151b*^*KO/KO*^ mutant mice in the ImPress protocols found no abnormal behavioural phenotype.

Given the strong statistical significance of profile comparisons (HHpred), and the concordance of known and predicted secondary structures and conserved active site residues (Fig. 1), we have shown that the FAM151 proteins are members of the PLC-like phosphodiesterase superfamily of enzymes. However, the remarkable substrate diversity of its phosphodiesterase homologues, means our computational analysis is unable to predict the substrate of the FAM151 family. Ultimately, identification of substrates and products will enhance our understanding of dendritic branching in nematode and retinal maintenance in mammals.

## Material and Methods

### Sequence analysis

The computational protein sequence analysis began by performing a JackHMMER iterative search^24^ beginning from the human FAM151B protein sequence, against the UniRef50 database^6^. The FAM151 family is widely distributed in animals, including nematodes (*C. elegans*; UniProt: O45879) and hexapods (*D. melanogaster*; UniProt: Q9VLU9). This reproduces the family phyletic distribution reported in Pfam (Family: DUF2181/PF10223)^25^. Next, we took advantage of profile-versus-profile (HHpred)^7^ to search PDB70 database for more divergent FAM151 homologues using as input a profile generated from the multiple protein sequence alignment of FAM151 family. The PDB70 database contains profile hidden Markov models (HMMs) for representative sequences, clustered to 70% maximum pairwise sequence identity to reduce redundancy, drawn from the PDB (Protein Data Bank)^26^.

### Mice

Mice were generated at MRC Harwell from ES cells with a targeted “knock-out first”, *tm1a*, mutation in Fam151b, and subsequently crossed with CRE recombinase expressing mice to generate mice with a LacZ insertion in intron 2 and a deletion of exon 3 (the allele is Fam151b^tm1b(EUCOMM)Hmgu^, hereafter Fam151b^KO^). The ES cells are of C57BL/6N origin, and the resulting mice initially maintained by crossing with C57BL/6NTac mice, and offspring selected for absence of Cre recombinase. The mutants were subsequently crossed with and maintained on a C57BL/6J background, and genotyped to ensure removal of the Crb1 mutation originating from the C57BL/6N strain.

*Fam151a*^*KO/KO*^ mice were made by microinjection of CRISPR gRNA targeting exon 1 and Cas9 protein into the pronucleus of fertilized eggs (Table 2). Sequencing identified a mouse with a 19 and 6 bp deletion surrounding a 16 bp section in exon 1. gRNAs were chosen based on low off-target predictions and founder mice were bred with C57BL/6J mice first before breeding on.

**Table 2:** gRNA used to produce Fam151a^KO/KO^mice.

Fam151a gRNA	GGGTGCCCTGATGCAGGGTT AGG

All animal work was approved by the University of Edinburgh internal ethics committee and was performed in accordance with the institutional guidelines under license by the UK Home Office.

### Immunohistochemistry

Mice were culled and eyes were enucleated and placed into Davidson’s fixative (28.5% ethanol, 2.2% neutral buffered formalin, 11% glacial acetic acid) for 1 hour (cryosectioning) or overnight (wax embedding). For cryosectioning eyes were removed from Davidson’s fixative and placed into 10%, 15% and 20% sucrose in PBS for 15 mins, 15 mins and overnight respectively. Eyes were then embedded using OCT cryopreservant and kept at −80 until sectioned. For wax preservation eyes were removed from Davidson’s fix and placed successively into 70% ethanol, twice in 70% 80% xylene, then 90% paraffin and finally twice in 100%; paraffin each for 45 mins.

Heamatoxylin and Eosin staining was performed on 8 µm paraffin tissue sections and imaged on a Zeiss Brightfield microscope.

Glial Fibrillary Acidic Protein (GFAP) antibody (ab7260, Abcam), RPE65 (ab13826, Abcam), Rhodopsin (MAB5356, Millipore), M/L-Opsin (AB5405, Millipore) were used on 8um frozen sections at 1:100 in blocking buffer (TBS with 0.1% triton x-100 and 4% donkey serum) and imaged on a Nikon A1R microscope.

For wholemount staining of RPE eyes were enucleated and immersed in 2% PFA for 3 minutes, and washed twice in PBS for 5 minutes. The rest of the retina was dissected from the RPE by removing the cornea and lens and carefully peeling the retinal tissue off to leave only the RPE attached to scleral tissue. Radial incisions were made in order to lay the RPE flat and methanol was added slowly and the tissue was placed at −20 °C for four hours. Staining was then performed on the RPE using the ZO-1 antibody (33–9100, Thermo Scientific) 1:100 in blocking buffer. All staining was performed on an n of 3 for each genotype.

For wholemount staining on embryonic skin at embryonic day 16.5 mice were collected and culled by schedule 1 approved methods. Limbs were removed and the body was placed in 4% PFA at 4 °C overnight. The bodies were then washed three times in PBS for five minutes at room temperature and transferred to 100% Methanol at −20 °C for 4 hours. The skin was then removed and rehydrated in Methanol/PBS-T (PBS with 0.2% triton X-100) mixtures of 75%, 50% and 25% for five minutes each. The skin was then stained with Neurofilament (2H3, DSHB) antibody 1:400 in blocking buffer.

### Electroretinography

All mice undergoing an ERG were dark adapted overnight prior to the procedure, and experiments were carried out in a darkened room under red light using an HMsERG system (Ocuscience). Mice were anesthetised using isofluorane and pupils were dilated through the topical application of 1% w/v tropicamide before being placed on a heated ERG plate. Three grounding electrodes were used subcutaneously (tail, and each cheek) and silver embedded electrodes were placed upon the cornea held in place with a contact lens. The standard International Society for Clinical Electrophysiology of Vision (ISCEV) protocol was used which recorded scotopic responses before a 10 min light adaption phase in order to record photopic responses^27^. 3 and 10 cd.s/m^2^ light intensity scotopic responses were used for analysis. Data was analysed using Graphpad Prism and compared by unpaired t-test with Welch’s correction.

### Western blot

Protein samples were ran on a 4–12% Tris-Bis gel (Invitrogen) and transferred to a PVDF membrane. Anti-α-tubulin (ab4074, Abcam) and anti-Fam151a (NBP2-13983, Novus Biologicals) were all diluted 1:1000 in TBS with 0.1% tween-20 and 5% Marvel and incubated overnight at 4 °C. Horseradish-peroxidase-conjugated anti-rabbit (NA934V, GE Healthcare Life Sciences) antibody was diluted 1:7000 in PBS with 0.1% tween-20 and 5% Marvel and incubated at room temperature for 1 h.
